# Comparative evaluation of bond strength of resin cements with and without 10-methacryloyloxydecyl dihydrogen phosphate (mdp) to zirconia and effect of thermocycling on bond strength – An *in vitro* study

**DOI:** 10.4317/jced.59324

**Published:** 2022-04-01

**Authors:** Gopala Abhishek, S. K. Vishwanath, Anoop Nair, Nagaranjani Prakash, Abhisikta Chakrabarty, Ashrith K. Malalur

**Affiliations:** 1 MDS. Post Graduate, Government Dental College and Research Institute, Bengaluru, Karnataka, India; 2 MDS. Associate Professors, Department of Prosthodontics, Government Dental College and Research Institute, Bengaluru, Karnataka, India; 3 BDS. Private Practitioners, Bharadwaj Dental Clinic, Bengaluru, Karnataka, India

## Abstract

**Background:**

To compare bond strength of resin cements with and without 10-Methacryloyloxydecyl Dihydrogen Phosphate (MDP) to zirconia and evaluate effect of thermocycling on bond strength.

**Material and Methods:**

Standardised test specimens were fabricated as per ADA specification 131. Each Zirconia specimen was mounted in autopolymerzing acrylic resin material. The specimens were divided into 2 groups: Group 1 – specimens bonded with resin cement containing 10-MDP and Group II - specimens bonded with resin cement without 10-MDP. Forty samples of resin cement cylinders were prepared with dimensions of 6mm height and 4mm diameter in line with ADA specification 27 were cured onto the zirconia surface of 10mm x10mm x5mm using customised moulds. Specimens from each cement group were further divided into 2 subgroups: Subgroup A – Specimens that were not thermocycled and Subgroup B – Specimens that were thermocycled. Specimens were then subjected to tensile bond testing by using a Universal testing machine, the data were analysed using independent sample t test for bond strength and paired t test for effect of thermocycling. Statistical analysis used: Data was subjected to normalcy test (Shapiro-wilk test). Data showed normal distribution. Hence parametric test paired t test were applied.

**Results:**

Paired t test revealed that the thermocycling affected the bond strength to zirconia. The highest bond strength was achieved for the resin cement with 10-MDP before thermocycling, whereas the lowest bond strength values were recorded for resin cement without 10-MDP after thermocycling.

**Conclusions:**

Resin cement with 10-MDP showed superior bond strength to Zirconia than resin cement without 10-MDP. Adhesive failure was predominant at Zirconia and resin cement interface. Thermocycling had a significant effect on the bond strength of resin cements to zirconia, showing decreased bond strength.

** Key words:**10-MDP, Tensile Strength, Zirconia.

## Introduction

Ceramics were ﬁrst used successfully in dentistry in 1774, at a time when tooth coloured replacements were fabricated from ivory, bone, wood, animal teeth, or expensive extracted teeth obtained from human “donors.” None of these biologically derived products was stable toward oral ﬂora or corrosive components of food and saliva. Ceramics immediately solved the issues of stained and decayed dentures (1).

The discovery of Zirconia by the German chemist Martin Heinrich Klaproth in 1789 and later was proposed for medical purposes in 1969 for concerned orthopaedic application as an emerging material for hip head replacement rather than titanium or alumina prostheses (2). Dr Charles Land patented the first Ceramic jacket crowns in 1903. The fracture strength was intensified, in early 1950s the porcelain restorations were strengthened with a metal substructure on which the porcelain was fused (3,4).

Rapid improvements of the all-ceramic restorations, combined with the employment of Computer-Aided Design (CAD)/Computer-Aided Manufacturing (CAM), has made digital dentistry increasingly favoured over the past decade. CAD/CAM systems are continuously developed and upgraded in prosthetic dentistry, and in association with zirconium oxide, is used primarily for the restoration of single crowns and fixed partial dentures (FPDs) and, dental implantology, as abutments or implants (5).

Bindl *et al*. reported that conventional luting failed to reduce fracture resistance of Zirconia-ceramic restoration. Surface treatment had no impact in relation with the adhesion (6). Presently the trebochemical treatment application is favoured as the surface treatment option for the ceramic restorations prior luting with resin cement. With advanced clinical trials and substantial studies, resin cementation appears one of the most favourable choices to achieve satisfactory adhesion and to enhance the mechanical features of Zirconia restorations (7).

This chemical coupling procedure is easy, non-invasive to ceramic substrates, and results in excellent bonding outcomes. Adding the phosphate ester monomer 10-Methacryloyloxydecyl Dihydrogen Phosphate (MDP) to bonding agents (primers or cement) appears to reinforce bond strength to zirconia because P-O-Zr chemical bonds are formed between MDP and zirconia (8). It was further proposed that integration of MDP-containing agents with airborne-particle abrasion surface roughening could generate a stronger bond to zirconia through chemical bonding and micromechanical interlocking (9).

Presently 10-MDP is the most widely-used phosphate ester monomer applied for the coupling of Y-TZP. Chemical bonding between 10-MDP and Y-TZP has been substantially studied. MDP has been incorporated into various bonding and luting products, such as primers, adhesives, and resins (10). Therefore, the purpose of this study was to evaluate the effect of MDP containing resin-based luting cement on the bond strength to Y-TZP and the impact of thermocycling on Bond Strength.

## Material and Methods

Institutional ethical committee clearance number GDCRI/IEC-ACM(2)/9/2018-19.

This *in vitro* study was conducted to compare the bond strength of resin cement with 10-Methacryloyloxydecyl Dihydrogen Phosphate (MDP) as its constituents against the regular resin cement to zirconia and evaluate the impact of thermocycling on bond strength.

-Fabrication of Standardised test specimens:

A wax pattern was prepared with dimensions of 10x10x05 mm using modeling wax. Later a mould was prepared using medium body elastomeric impression material. Die stone was poured onto to the prepared surface. The final block was sandpapered and transferred to the extra oral scanner. A Zirconia block (Ceramill, Amann Girrbach AG

Herrschaftswiesen 6842 Koblach | Austria) was used for the study. Forty Zirconia specimens were machined from a CAD/CAM Milling System (Ceramill® motion 2, Amann Girrbach AG Herrschaftswiesen 6842 Koblach | Austria).

-Surface treatment of the Zirconia blocks.

The forty specimens were surface treated with sand blasting.

(50 µm aluminium oxide particles for 2 minutes under 2 bar pressure, at a distance of 10 mm)

•Preparation of Samples of Group 1: Panavia 2.0 

(Kuraray America, Inc. Dental Division, 32 Old Slip Floor 7 New York, NY 10005)

Surface of 20 Zirconia specimens were bonded with resin cement with 10-MDP. Equal amounts of ED Primer II A&B were incorporated. Later applied to the fabricated specimen. Equal amounts of A&B mixed for 20 seconds. Mixture of this paste was applied into the customized molds.

•Preparation of Samples of Group 2: Maxcem Elite 

(KERR CORPORATION, 200 S, Kraemer Blvd, Building E2 Brea, CA 92821)

Surface of 20 Zirconia blocks was bonded with resin cement without 10-MDP. The dual barrel syringe with auto mix tips and angulated dispensing tips were used to deliver the desired amount of the cement.

-Fabrication of test specimens:

All the 40 Zirconia blocks were embedded in acrylic resin (DPI RR Cold cure, Commercial Union House, 9 Wallace Street, Fort, Mumbai, India) blocks (10 mm length × 10 mm breadth × 5 mm height) with the surface treated side of block exposed. To fabricate standardized resin cement cylinders which were bonded onto the ceramic surfaces, customized clear vinyl plastic tubes, 4 mm in diameter and 6 mm in height were used. To facilitate the testing of tensile bond strength between Zirconia and resin cement, Orthodontic wire of 0.6 mm diameter was placed through the molds. Resin cement was slightly overfilled in the molds and was then positioned firmly on the Zirconia bonding sites. The resin cement cylinders were then polymerized using a light-polymerizing unit, with the light angulated approximately 45 degrees from the junction of the Zirconia bonding sites and resin cement cylinders. Two cycles of 20 seconds light polymerization were carried around the circumference of the composite resin cylinder to strengthen the bond.

-Thermocycling of the samples:

Each group was subdivided into 2 subgroups A & B of 10 Samples each. After cementation, 10 samples of each group were subjected to thermocycling in two different thermal baths with temperature maintained at 5ºC and 55ºC using distilled water. A temperature regulating button and thermometer was used to record temperature fluctuation. Each sample was exposed to thermocycling for a period of 15s at 5ºC and 55ºC with 15s interval between each cycle and total of 5000 Temperature cycles.

**Figure 1:**
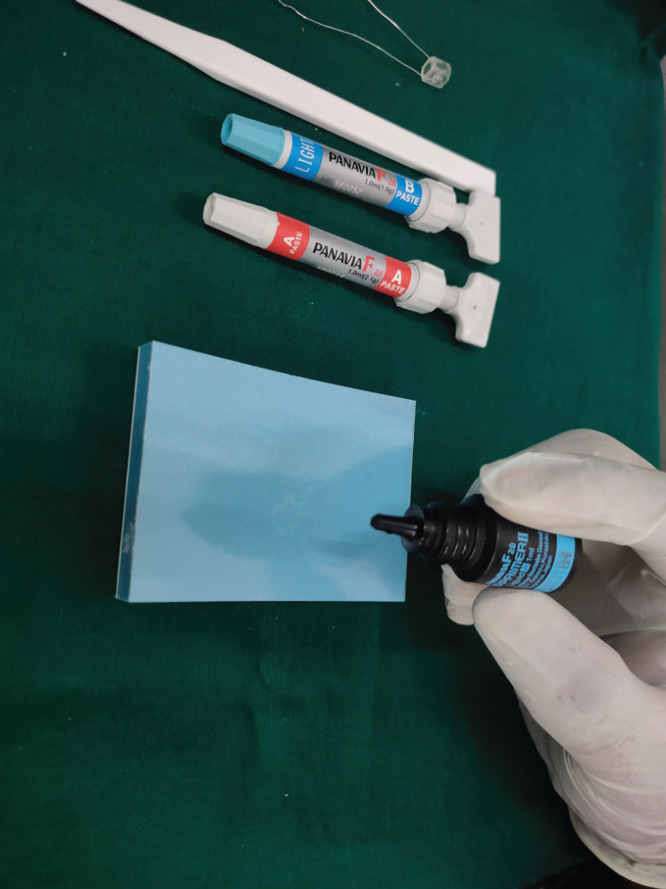
Primer Application of Panavia 2.0.

Testing of specimens:

Each specimen was mounted on the lower fixture of a universal testing machine (MultiTest 10 -i, Mecmesin, UK), and the orthodontic wire was connected to the upper fixture (Fig. 1). The bonded composite resin cylinders were then subjected to a tensile force at a crosshead speed of 2 mm/min until fracture occurred, and tensile bond strengths were calculated using the formula:

σ = P/A, where σ is the tensile bond strength (MPa), P is the maximum force (N), and A is the interfacial area (mm2) and date recorded in computer.

**Figure 2:**
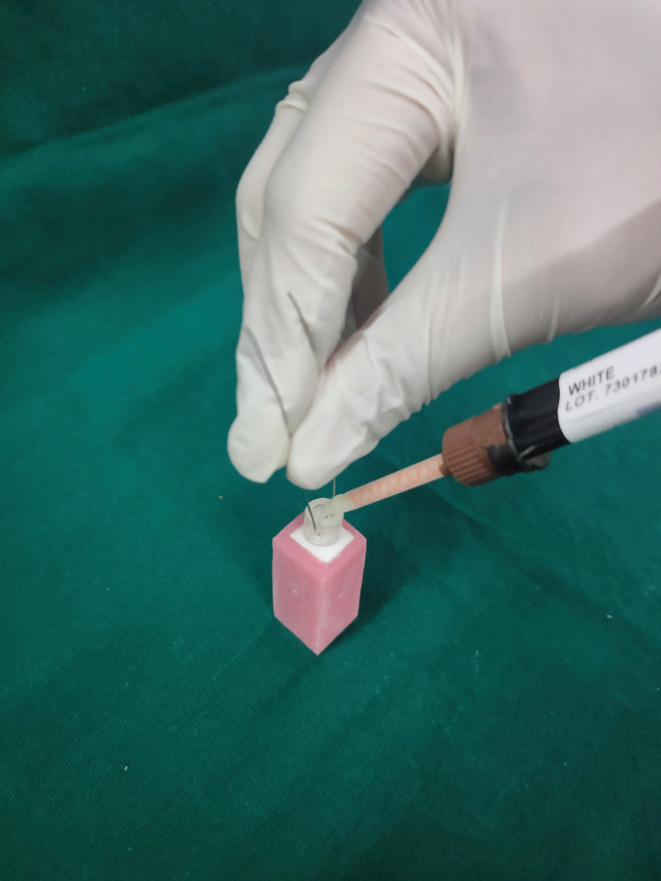
Kerr Maxcem applied into the Test Specimen.

**Figure 3:**
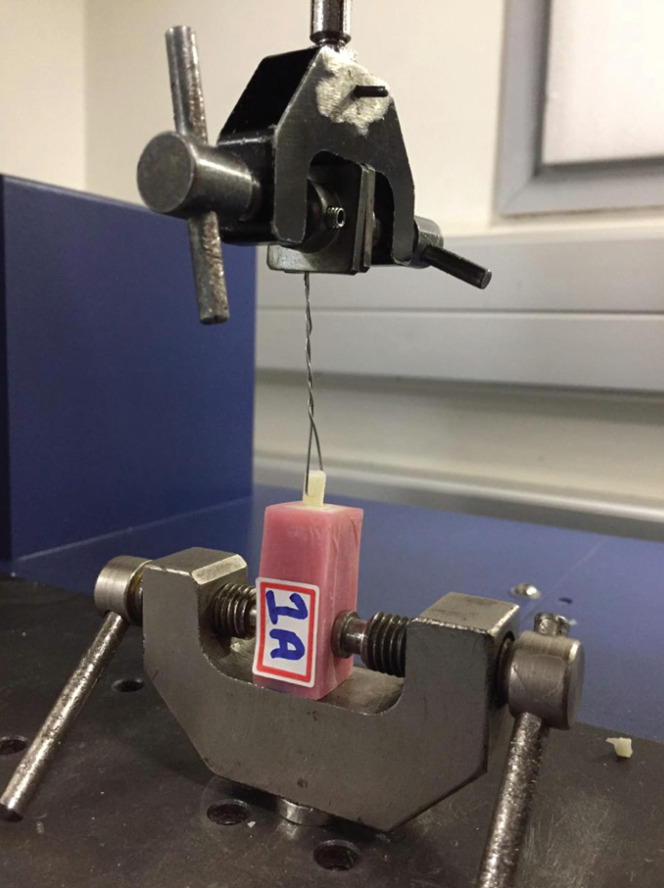
Testing Specimen mounted onto Universal Testing Machine.

-Site of Tensile Failure:

For all the test specimens, the interface where fracture occurred was classified as either cohesive or adhesive in nature. The test specimen which fractured at the interface was examined under magnification to determine whether the fracture was adhesive or cohesive. A layer of resin cement present on the Zirconia surface after fracture was inferred as cohesive failure. From the total samples of 40, adhesive failures (Fig. 2) were evident in 37 samples and 3 were cohesive failures (Fig. 3).

## Results

In this study, tensile strength of 20 resin cement with 10-MDP and 20 without 10-MDP samples, (10 before thermocycling and 10 after thermocycling) were tested. (Table 1).

**Table 1:**
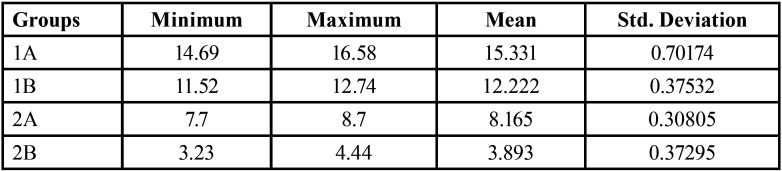
Mean tensile strength.

## Discussion

Aesthetically driven approach has caused dental professionals and patients to opt for the use of metal‑free restorations in prosthodontics. The substantial advancement associated with leucite, lithium disilicate, zirconia, and alumina‑reinforced ceramics has allowed substitution of metallic infrastructures in diverse clinical situations, due to their high flexural and compressive strength (11,12). The present study was undertaken to compare the tensile bond strength of two different resin cements (With 10-MDP and without 10-MDP as its constituents) bonded to Zirconia; before and after thermocycling. There was no significant difference in the tensile bond strength of the tested groups, thus the results of this study led to the rejection of null hypothesis.

The results in this study showed that the mean bond strength of Panavia 2.0 bonded to Zirconia with and without thermocycling was, respectively, 15.331 MPa and 12.222 MPa. The mean bond strength of Maxcem bonded to Zirconia with and without thermocycling was, respectively, 8.165 MPa and 3.893 MPa. While comparing two resin cements, it was observed that a statistically significant (*P* > 0.05) difference in mean tensile bond strength was observed.

Panavia 2.0 resin cement presented highly significant results than Kerr Maxcem. The disparity in the bond strength observed could be due to difference in the chemical composition of the two types of cement used. Panavia 2.0 is composed of methacrylate monomers containing phosphoric acid groups which are all frequently used cross‑linkers in adhesive systems (13). Phosphoric acid methacrylates, the main constituent of the Panavia 2.0 are stated to react with the hydroxyapatite of the hard tissue like enamel when these monomers dissociate into methacrylate and the acidic phosphoric acid in an aqueous solution (14).

The impact of silane incorporated in a universal multimode adhesive might be limited. In general, so‑called universal primers/adhesives achieve more durable bonding to zirconia than to lithium disilicate (15). Thermal cycling was done for half of the samples of each group to evaluate the effect of changing intraoral conditions on the shear bond strength of ceramics. In this study, the samples were subjected to 5000 cycles with bath temperatures of 5°C and 55°C with a dwell time of 15 s according to ISO standardization (16).

**Figure 4:**
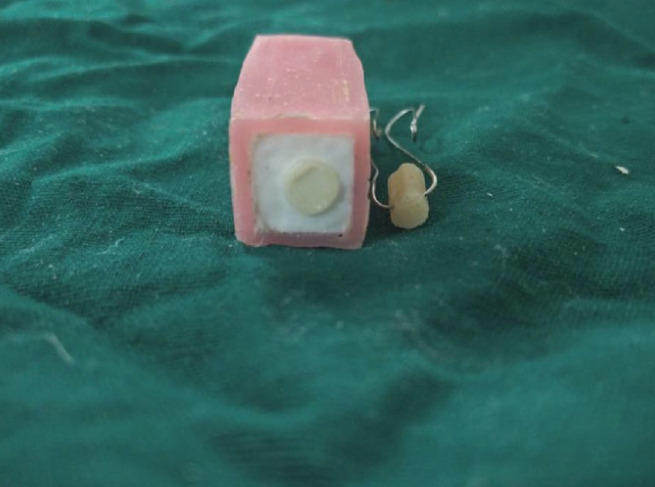
Zirconia Surface after Tensile Bond Testing (Adhesive Failure).

The results showed that the difference between the mean tensile bond strength of Zirconia with Panavia 2.0 and Maxcem Elite with and without thermocycling was statistically significant difference. Failure analysis revealed that failures were predominantly adhesive nature in the resin cements (Fig. 4).

The main disadvantage of such *in vitro* studies is that the oral conditions cannot be simulated. Subjecting the specimens to active loading in artificial saliva prior to testing may closely resemble intraoral conditions with respect to hydrolytic degradation of the bond due to pH changes of saliva and the effect of temperature change in the oral cavity. Furthermore, other clinically relevant factors such as conFiguration of crown preparation, dentinal tubular fluid movement, pulpal pressure, remaining dentin thickness, and type of dentin should be considered when testing adhesive systems *in vitro* (17,18).

## Conclusions

Considering the limitations of this study, conclusion can be summarised as,

1. The bond strength of resin cements with 10-MDP as its constituent is much better with zirconia than that of without 10-MDP.

2. Thermocycling has significant effect on the bond strength between resin cements with and without 10-MDP and zirconia, decreasing the bonding efficiency of the resin cements.
